# Investigation of the factors associated with circulating soluble CD36 levels in patients with HCV-related chronic liver disease

**DOI:** 10.1186/1758-5996-5-51

**Published:** 2013-09-09

**Authors:** Takashi Himoto, Joji Tani, Hisaaki Miyoshi, Asahiro Morishita, Hirohito Yoneyama, Kazutaka Kurokohchi, Michio Inukai, Hisashi Masugata, Fuminori Goda, Shoichi Senda, Reiji Haba, Masaki Ueno, Genji Yamaoka, Tsutomu Masaki

**Affiliations:** 1 Department of Integrated Medicine, Kagawa University School of Medicine, 1750-1, Ikenobe, Miki-Cho, Kagawa 7610-79, Japan; 2 Department of Gastroenterology and Neurology, Kagawa University School of Medicine, Kagawa, Japan; 3 Department of Diagnosis Pathology, Kagawa University School of Medicine, Kagawa, Japan; 4 Department of Pathology and Host Defense, Kagawa University School of Medicine, Kagawa, Japan; 5 Department of Clinical Laboratory, Hospital of Kagawa University School of Medicine, Kagawa, Japan

**Keywords:** CD36, Hepatic steatosis, Hepatitis C virus, Insulin resistance, Oxidized low-density lipoprotein

## Abstract

**Background:**

CD36, a class B scavenger receptor, participates in the pathogenesis of metabolic dysregulation such as insulin resistance, hepatic steatosis, and atherosclerosis. Persistent hepatitis C virus (HCV) infection often evokes these metabolic abnormalities. The primary purpose of this study was to investigate the role of CD36 in the pathogenesis of insulin resistance and hepatic steatosis caused by chronic HCV infection.

**Methods:**

Forty-five patients with HCV-related chronic liver disease (CLD-C) were enrolled in this study. CD36 expression in the liver specimen was examined by an immunohistochemical procedure. The concentrations of circulating soluble form of CD36 (sCD36) and oxLDL were determined by the enzyme-linked innunosorbent assay. Insulin resistance was estimated by the values of HOMA-IR.

**Results:**

Moderate to extensive hepatic CD36 expression was observed in the sinusoids of all enrolled CLD-C patients. CD36-positive sinusoids appeared to be identical to Kupffer cells. The severity of CD36 expression in the hepatic sinusoids was significantly correlated with the sCD36 level in sera of patients with CLD-C. The serum sCD36 levels were significantly correlated with body mass index and serum oxLDL levels in those patients. However, the serum sCD36 concentrations were independent of the values of HOMA-IR and the severity of hepatic steatosis.

**Conclusions:**

These data suggest that the serum sCD36 levels reflect the severity of CD36 expression on the Kupffer cells in patients with CLD-C, and that the serum sCD36 levels were associated with obesity, although the levels were independent of insulin resistance and hepatic steatosis in those patients.

## Introduction

An estimated 180 million people in the world are chronically infected with hepatitis C virus (HCV). The virus induces a spectrum of chronic liver diseases from chronic hepatitis to liver cirrhosis, and ultimately to hepatocellular carcinoma (HCC) [1].

Persistent HCV infection often evokes numerous kinds of metabolic abnormalities, including insulin resistance, hepatic steatosis, dyslipidemia and iron overload [2-4]. These metabolic abnormalities, which are induced by the direct actions of HCV itself [5] and/or through various host factors [2-4], are involved in the pathogenesis of chronic liver damage. Insulin resistance, hepatic steatosis and iron overload have also been considered to be predictive risk factors refractory to antiviral treatments like pegylated interferon (PEG-IFN) alone or PEG-IFN plus ribavirin in patients with chronic hepatitis C (CH-C) [6-8]. Moreover, insulin resistance seems to be associated with not only hepatic fibrosis but also with carcinogenesis in those patients [9,10]. Impairment of insulin receptor substrate-1 (IRS-1) by HCV core protein directly or through a variety of cytokines and trace elements may account for insulin resistance in those patients [11]. We previously revealed that zinc and selenium deficiency resulted in impaired insulin signaling in patients with HCV-related chronic liver disease (CLD-C) [12,13].

Recent studies have revealed that CD36, also known as fatty acid translocase (FAT), is expressed on the surface of a variety of cells including adipocytes, skeletal muscle cells, platelets, monocytes, endothelial cells, and macrophages as a receptor for oxidized low-density lipoprotein (oxLDL) [14,15]. CD36 has been proposed to be a surrogate of macrophage activation and inflammation [16]. Moreover, it has been widely studied that CD36 plays a pivotal role in the metabolic dysregulation including obesity, insulin resistance and atherosclerosis [14,17]. Therefore, CD36 expression on the surface of monocytes and macrophages is up-regulated by oxLDL in patients with type 2 diabetes mellitus (DM) [18]. CD36 also has been considered to contribute to the progression of hepatic steatosis [19,20]. In contrast, one study has suggested that CD36 deficiency, characterized by the absence of CD36 expression in peripheral platelets alone or both platelets and monocytes, is associated with insulin resistance in patients with coronary heart diseases [21].

CD36 is in the category for the class B scavenger receptor [14], and the human scavenger receptor class B type I (SR-BI) has been shown to be a novel candidate receptor for HCV of genotypes 1a and 1b [22]. Miquilena-Colina and colleagues demonstrated that the expression of CD36 was up-regulated in the liver of patients with nonalcoholic steatohepatitis (NASH) and patients infected with HCV of genotype 1 [23].

More recently, a soluble form of CD36 (sCD36) was identified in human plasma as a novel biomarker for type 2 DM [24]. Handberg and colleagues proposed that higher levels of sCD36 predicted the development of type 2 DM irrespective of age, gender and obesity [25]. They also speculated that sCD36 might be circulating in microparticles derived from monocytes, platelets, or endothelial cells [25].

The primary purposes of this study were to elucidate the role of CD36 in the pathogenesis of insulin resistance caused by persistent HCV infection, to investigate the changes in the circulating sCD36 and oxLDL levels by antiviral treatments, and to determine whether or not CD36 deficiency is dependent on insulin resistance in patients with CLD-C.

## Methods

### Study population

Forty-five non-diabetic patients with CLD-C, all of whom were admitted in the Hospital of Kagawa University School of Medicine, and had detectable serum HCV-RNA by polymerase chain reaction (PCR) and showed histological findings compatible with chronic hepatitis or liver cirrhosis, were randomly selected for participation in this study (Table 1). Eight cases of normal healthy controls (NHCs) were also assigned to the comparison group. Blood samples were drawn in the morning with fasting before anti-viral treatments. This clinical trial was approved by the ethics committee of Kagawa University School of Medicine. Fully informed consent was obtained from each participant.

**Table 1:** Clinical characteristics of the enrolled patients with CLD-C

	
Age (y.o.)	56.3 ± 9.2(34–74)
Gender (Male/Female)	25/20
BMI	23.3 ± 3.4(17.0-31.9)
Staging (F_1_/F_2_/F_3_/F_4_)	16/16/7/6
Steatosis (grade 0/1/2)	26/13/6
HCV genotype (1b/2a/2b)	28/13/4
Loads of HCV-RNA (KIU/ml)	2537 ± 2766 (48–10000)
Values of HOMA-IR	2.18 ± 1.40 (0.51-5.53)

### Laboratory assessments

The serum alanine aminotransferase (ALT) levels were determined by the standard laboratory technique. The circulating sCD36 and oxLDL concentrations were determined by a commercially available enzyme-linked immunosorbent assay (ELISA) kit (Cusabio Biotech Co. Ltd, Wuhan, China). The minimums detectable dose of sCD36 and oxLDL are 4 pg/ml and 0.039 μmol/ml, respectively. Quantitative detection of serum HCV-RNA was performed by the Amplicor-HCV monitor assay (Roche Molecular Diagnostics, Tokyo, Japan) [26] or COBAS TaqMan HCV assay (TaqMan) (Rosche Diagnostics, Tokyo, Japan) [27]. The values measured by the TaqMan assay were adjusted to those determined by the Amplicor-HCV monitor assay. The HCV-genotype was determined by the HCV-RNA genotyping assay system (Home Brew SRL Inc., Tokyo, Japan) [28].

In addition, insulin resistance was determined by the values of the Homeostasis Model for Assessment of Insulin Resistance (HOMA-IR) method using the following equation: HOMA-IR = Fasting insulin (μU/ml) × fasting glucose (mg/dl)/405. Insulin resistance was defined as the values of HOMA-IR exceeding 2.0. Body mass index (BMI) was estimated as a hallmark of obesity. Obesity was defined as BMI over 25.0.

### Histological assessments

Liver tissue specimens were obtained by liver biopsy under the guidance of ultrasound, using 16-gauge needles, before antiviral treatments. The tissue samples were fixed in 10% formalin and embedded in paraffin. The tissue sections were stained with hematoxylin and eosin for morphological evaluation. Hepatic fibrosis was evaluated in accordance with the New Inuyama Classification system [29], the standard criterion for the histological assessment of chronic hepatitis in Japan. Briefly, the staging of hepatic fibrosis was scored as follows: F_0_, no fibrosis in the liver specimen; F_1_, portal expansion; F_2_, bridging fibrosis; F_3_, bridging fibrosis with lobular distortion; and F_4_, liver cirrhosis. The severity of hepatic steatosis was graded on the basis of the classification proposed by Brunt and colleagues [30]. In brief, steatosis observed in none, less than 33%, 33-66%, or more than 66% of hepatocytes was defined as grade 0, 1, 2, or 3, respectively.

### Immunohistochemical procedure

The expression of CD36 protein in the liver specimens of the enrolled patients with CLD-C was examined by an immunohistochemical procedure. In brief, each tissue section was deparaffinized, then washed by phosphate-buffered saline (PBS) and incubated with mouse anti-human monoclonal antibody to CD36 (OriGene Technology, Inc, Rockville, MD, USA) as a primary antibody. After washing with PBS, the tissue section was reacted with biotinylated goat anti-mouse polyclonal antibody. Thereafter, color was developed with diaminobenzine (DAB). Counter staining was performed with hematoxylin. The degree of hepatic CD36 expression was classified into three categories: none, moderate and extensive.

For dual immunofluorescence staining, the tissue sections were incubated with mouse anti-CD14 antibody (1:400, Santa Cruz Biotech, CA, USA), followed by incubation in Alexa Fluor 594-conjugated anti-mouse IgG (Molecular Probes, Eugene, OR, USA). In addition, the sections were incubated with rabbit anti-CD36 antibody (1:50, Novus, Littleton, CO, USA), followed by incubation in Alexa Fluor 488-conjugated anti-rabbit IgG (Molecular Probes). Then, the sections were incubated in Monomeric Cyanine Nucleic Acid Stains (TO-PRO-3, Molecular Probes), which was diluted in phosphate-buffered saline (PBS). The fluorescent signals were viewed under a confocal microscope (Carl Zeiss LSM700, Oberkochen, Germany). As a control experiment, we performed an identical immunohistochemical procedure by omitting the primary antibodies.

### Diagnosis of CD36 deficiency

Fluorescein isothiocyanate (FITC)-conjugated anti-human monoclonal antibody against CD36 (Beckman Coulter, KK, Shizuoka, Japan) was used to detect CD36 in immunofluorescent flow cytometry. The signal from the anti-CD36 was gated with phycoerythrin (PE)-conjugated anti-human monoclonal antibody against CD41 (Beckman Coulter KK, Shizuoka,Japan), a specific marker for platelets, or PE-conjugated anti-human monoclonal antibody against CD14 (Beckman Coulter KK, Shizuoka, Japan), a specific marker for monocytes. For the platelet experiment, platelet-rich plasmas were analyzed.

CD36 deficiency was defined as the absence of CD36 molecules on the surface of peripheral monocytes and platelets (type 1), or peripheral platelets alone (type 2), by flow cytometry analysis.

### Statistical analyses

Data values are represented as means ± standard deviations (SDs). The Mann–Whitney *U*-test was used for the comparison of variables between two groups, while the Bonferroni/Dunn method was carried out for comparisons among three groups. The relationships between quantitative variables were analazed by Pearson’s test. The paired *t*-test was used to compare the valuables before and at the end of antiviral treatments. P values less than 0.05 were considered to indicate significance.

## Results

### Clinical characteristics of the enrolled patients with CLD-C

As shown in Table 1, the ages at entry in the enrolled patients ranged from 34 to 74 years old (y.o.). Among those patients, 25 were male, and 20 were female. The average BMI in the enrolled patients was 23.3 ± 3.4. Sixteen of 45 (35.6%) patients with CLD-C were in the category of obesity. The severity of hepatic fibrosis in the enrolled patients was evaluated as follows: F_1_ in 16 patients, F_2_ in 16, F_3_ in 7, and F_4_ in 6. In the light of hepatic steatosis, 26 patients with CLD-C had no hepatic steatosis (grade 0), while 13 and 6 patients had hepatic steatosis grades 1 and grade 2, respectively. None of those patients had grade 3 hepatic steatosis. HCV-genotypes of the enrolled patients were 1b in 28 patients, 2a in 13 patients, and 2b in 4 patients. The loads of HCV-RNA in those patients ranged from 48 to 10,000 KIU/ml. The mean value of HOMA-IR in the enrolled patients was 2.18 ± 1.40. Nineteen of 45 (42.2%) patients with CLD-C fulfilled the criterion for insulin resistance.

In contrast, 3 males and 5 females were in the group of NHCs. Mean age in the NHC group was 43.8 ± 6.2 y.o. The BMI in each NHC case was all less than 25.

### Hepatic CD36 expression

**Figure 1:**
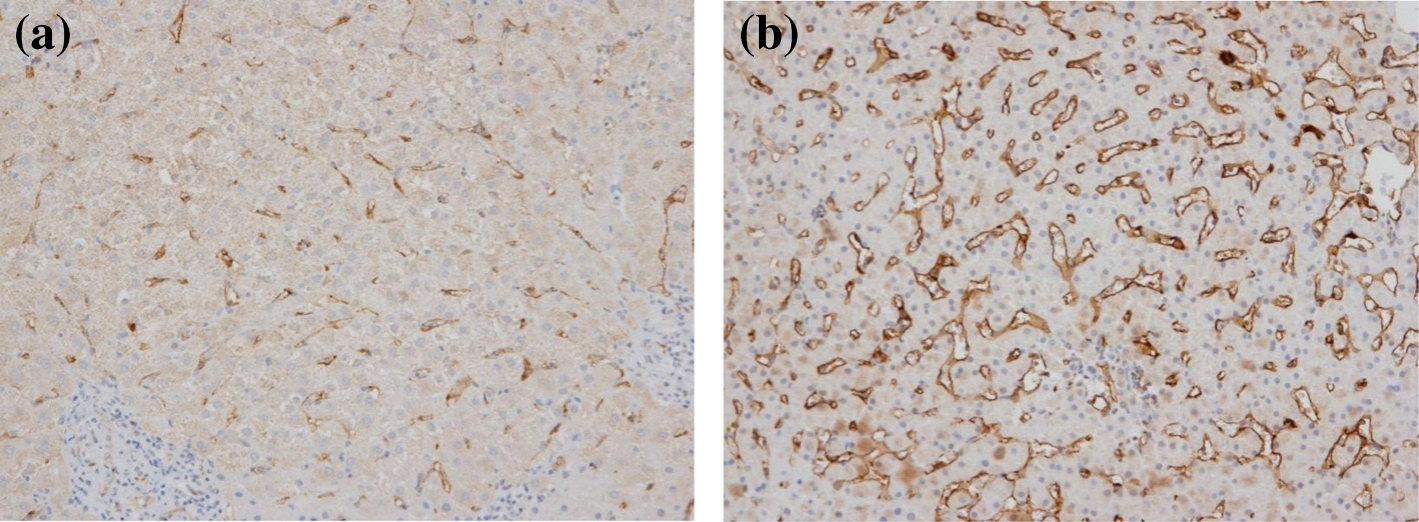
**Representative figures of the hepatic CD36 expression by an immunohistochemical staining. (a)** Moderate and **(b)** extensive degrees of CD36 protein were observed in the sinusoids. (original magnification × 100).

**Figure 2:**
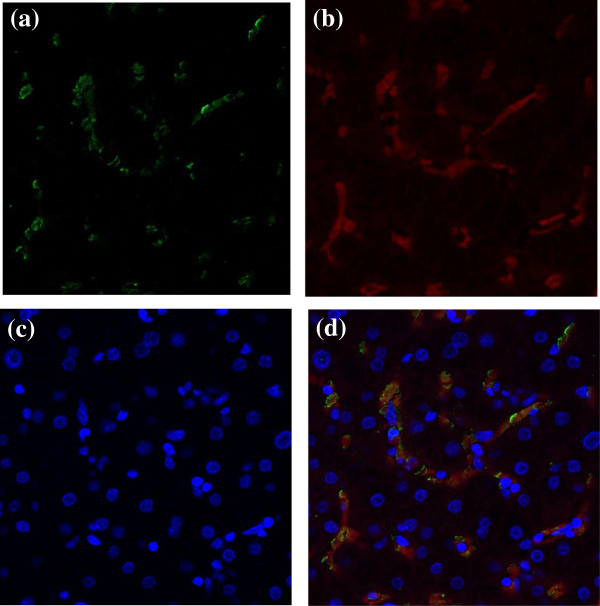
**Representative figures of dual staining with anti-CD14 and anti-CD36 in the liver specimens. (a)** immunohistochemical staining with anti-CD36 **(b)** immunohistochemical staining with anti-CD14, **(c)** nuclear staining with DAPI, **(d)** dual staining of anti-CD14 with anti-CD36 (original magnification × 100).

The localization and severity of CD36 expression in liver specimens of the 38 enrolled patients with CLD-C were examined by immunohistochemical techniques. All of the liver specimens had some expression of CD36 protein in the sinusoids: moderate expression in 19 patients and extensive expression in the other 19 (Figure 1a and 1b). The CD36-positive sinusoids were stained with anti-CD14, suggesting that Kupffer cells were candidates for CD36-positive sinusoids (Figure 2). However, none of the patients had CD36 expression in the hepatocytes.

### Correlation between the severity of CD36 expression in the liver and the circulating sCD36 concentration

**Figure 3:**
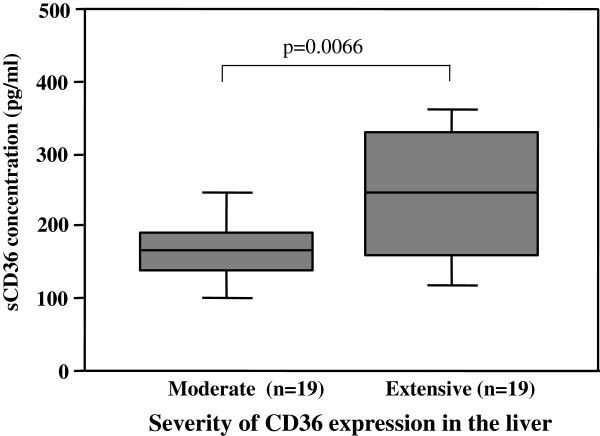
**Relationship between hepatic CD36 expression and serum sCD36 level.** Mean serum sCD36 level in the group with extensive hepatic expression of CD36 was significantly higher than that in the group with moderate hepatic expression of CD36.

The serum sCD36 levels were compared between the groups with moderate and extensive CD36 expressions in the liver. As shown in Figure 3, the mean circulating sCD36 level was significantly higher in the group with extensive hepatic CD36 expression than that in the group with moderate hepatic CD36 expression (250.1 ± 96.8 vs 167.2 ± 54.6 pg/ml, p = 0.0066). This result indicated that the circulating sCD36 might reflect the severity of CD36 expression in the hepatic sinusoids of the enrolled patients with CLD-C.

### Correlation between the circulating sCD36 concentrations and clinical parameters

**Figure 4:**
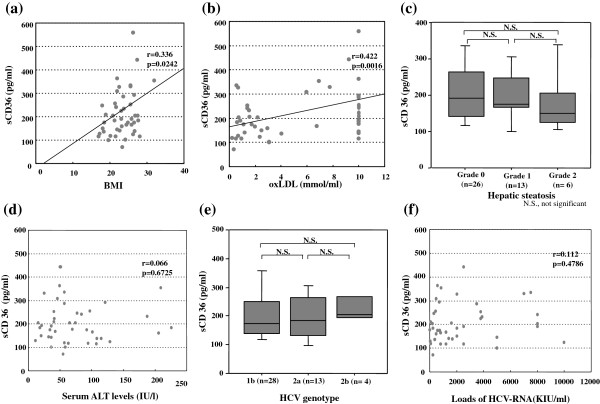
**Relationship between circulating sCD36 level and BMI, serum oxLDL level, severity of hepatic steatosis, serum ALT level, HCV-genotype or load of HCV-RNA. (a**,**b)** Serum sCD36 levels were roughly correlated with BMI or serum oxLDL levels. **(c**,**e)** Serum sCD36 levels were not associated with the severity of the hepatic steatosis or HCV-genotypes. The bars represent the maximum and minimum levels. The boxes represent the values within the 25th and 75th percentiles. The horizontal bars represent the medians. **(d**,**f)** Serum sCD36 levels were independent of serum ALT levels and loads of HCV-RNA.

The relationships between serum sCD36 levels and oxLDL, ALT, BMI or hepatic steatosis were investigated in patients with CLD-C. The serum sCD36 levels were roughly correlated with BMI in the enrolled patients (r = 0.336, p = 0.0242, Figure 4a). The serum sCD36 levels were also associated with serum oxLDL levels in those patients (r = 0.422, p = 0.0016, Figure 4b). However, the circulating sCD36 concentrations were independent of the severity of hepatic steatosis (Figure 4c) and the serum ALT levels (Figure 4d) in the enrolled patients. Neither HCV-genotypes nor loads of HCV-RNA were correlated with the circulating sCD36 concentrations (Figure 4e and 4f).

### Comparisons of the circulating sCD36 and oxLDL concentrations between CLD-C patients and NHC

**Figure 5:**
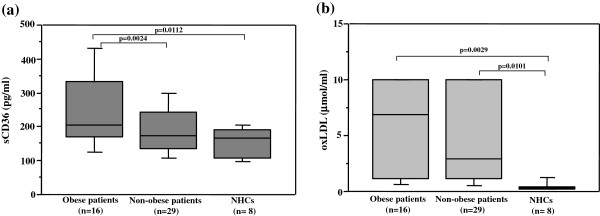
**Comparisons of serum sCD36 and oxLDL levels between patients with CLD-C and NHCs. (a)** The serum sCD36 levels were significantly higher in obese patients with CLD-C than in NHCs. Obese patients also had significantly higher serum sCD36 levels than non-obese patients. **(b)** The serum oxLDL levels were similarly higher in obese patients than in NHCs. However, no significant difference in the serum oxLDL level was found between obese and non-obese patients.

The serum sCD36 and oxLDL levels were compared between CLD-C patients and NHCs. Patients with CLD-C were divided into obese and non-obese groups, because the serum sCD36 levels were dependent on obesity. Obese patients with CLD-C had significantly higher circulating sCD36 concentrations than those in NHCs (254.7 ± 123.5 vs 153.7 ± 44.6 pg/ml, p = 0.0112, Figure 5a). The serum sCD36 levels were also higher in obese patients than in non-obese patients (254.7 ± 123.5 vs 190.5 ± 73.0 pg/ml, p = 0.0241, Figure 5a). Similarly, the serum oxLDL levels in obese patients with CLD-C were significantly higher than those in NHC (5.54 ± 4.15 vs 0.46 ± 0.44 μmol/ml, p = 0.0029, Figure 5b). However, no significant difference in serum oxLDL levels was found between obese and non-obese CLD-C patients (5.54 ± 4.15 vs 4.46 ± 3.97 μmol/l, p = 0.3587, Figure 5b).

### Correlation between insulin resistance and the circulating sCD36 or ox LDL concentrations

**Figure 6:**
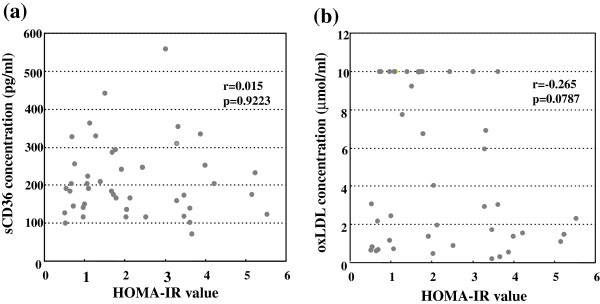
**Relationship between the values of HOMA-IR and serum (a) sCD 36 levels or (b) oxLDL levels in patients with CLD-C.** The values of HOMA-IR were not associated with serum sCD36 and oxLDL levels.

Next, we examined the relationshisp between insulin resistance and the circulating sCD36 or oxLDL levels in patients with CLD-C. No significant correlations were apparent between the circulating sCD36 concentrations and the values of HOMA-IR in those patients (r = 0.015, p = 0.9223, Figure 6a). The serum oxLDL levels were also independent of the values of HOMA-IR in those patients (r = -0.265, p = 0.0787, Figure 6b).

### Changes in the circulating sCD36 and oxLDL concentrations by antiviral treatments

The levels of circulating sCD36 and oxLDL were analyzed before and at the end of antiviral treatments such as pegylated interferon (PEG-IFN) alone or PEG-IFN plus ribavirin in eleven patients with chronic hepatitis C (CH-C), because these 11cases had paired serum samples before and the end of antiviral treatments. Among the patients with CH-C, 6 patients were genotype 1b, 3 patients were genotype 2a, and 2 patients were genotype 2b. Nine of 11 patients with CH-C acquired sustained virological response (SVR).

**Figure 7:**
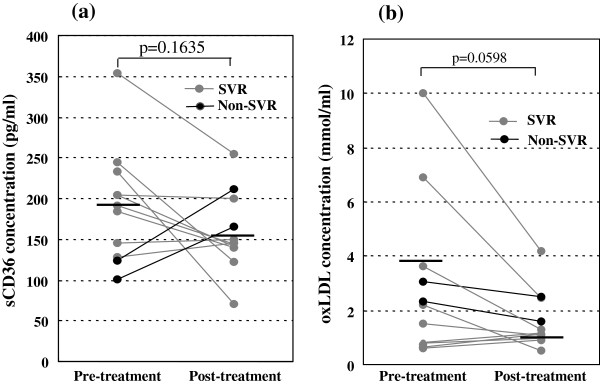
**Changes in the serum (a) sCD36 and (b) oxLDL levels by the antiviral treatments.** Serum sCD36 and oxLDL levels tended to be decreased by antiviral treatments. The horizontal bars represent the averages.

The serum sCD36 levels in the 9 CH-C patients with SVR were significantly decreased by the PEG-IFN-based treatments (209.8 ± 65.7 vs 148.5 ± 43.8 pg/ml, p = 0.0212), while the sCD36 levels in the other 2 CH-C patients without SVR were increased. Therefore, the circulating sCD36 concentrations at the end of the treatments tended to be decreased (192.1 ± 70.9 vs 155.7 ± 43.6 pg/ml, p = 0.1635, Figure 7a) in 11 patients with CH-C. The serum oxLDL levels also tended to be decreased by antiviral treatments (2.95 ± 2.99 vs 1.61 ± 1.06 μmol/ml, p = 0.0598, Figure 7b).

### Prevalence of CD36 deficiency

**Figure 8:**
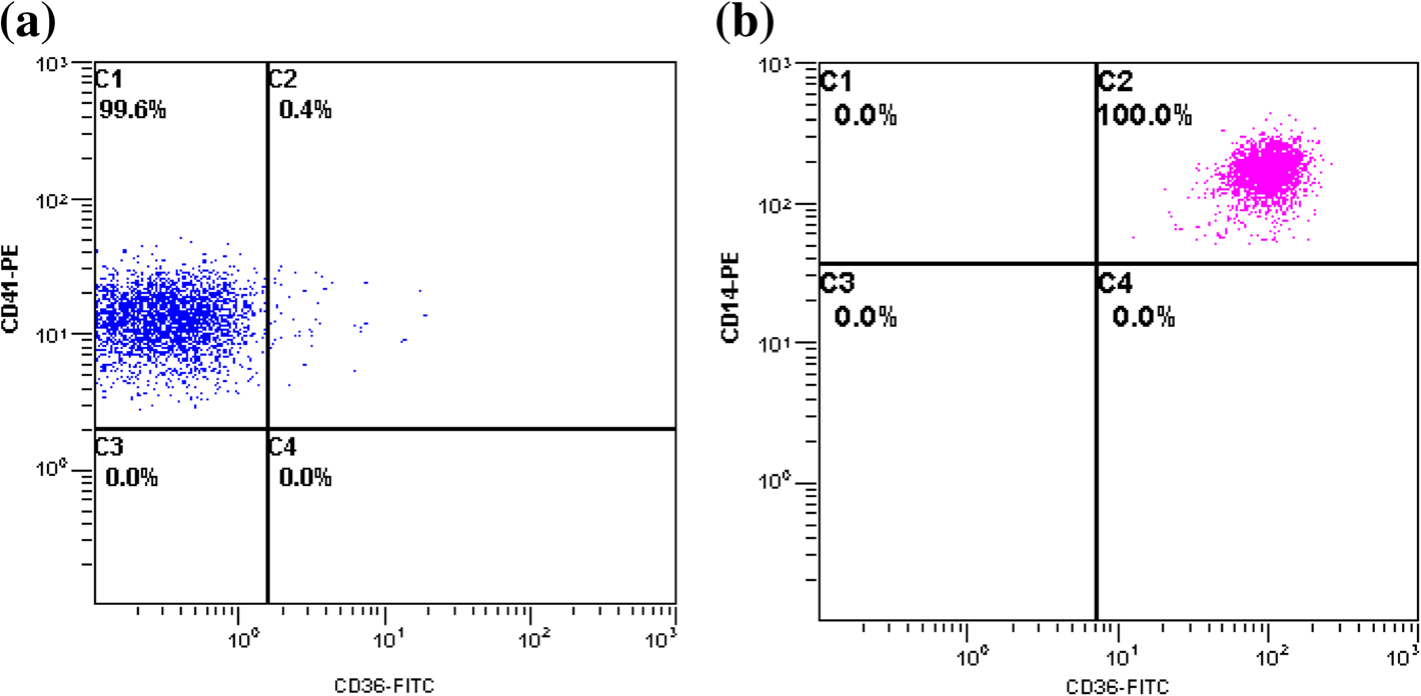
**Representative figure of CD36 deficiency type 2 by flow cytometry. (a)** A lack of CD36 expression on the surface of peripheral platelets was observed. **(b)** CD36 was normally expressed on the peripheral monocytes.

Peripheral blood was collected from 20 of the 45 patients with CLD-C. One of the 20 (5.0%) patients with CLD-C lacked CD36 expression on the surface of the peripheral platelets (Figure 8); this patient was diagnosed as CD36 deficiency type 2. He was infected with HCV of genotype 2a. The value of HOMA-IR in the patient was 1.68, indicating that the patient was not associated with insulin resistance. None of the patients with CLD-C fulfilled the category of CD36 deficiency type 1.

## Discussion

This study demonstrated that hepatic expression of CD36 was observed on the hepatic sinusoids of patients with CLD-C, and that the expression of CD36 in the sinusoids was colocalized with CD14 in patients with CLD-C, suggesting that CD36-positive sinusoids were identical to Kupffer cells. Imajo and colleagues documented CD14-positive Kupffer cells in the experimental model of steatosis [31]. The present study also revealed that the severity of CD36 expression generally paralleled the circulating sCD36 concentrations in patients with CLD-C. Alkhatatbeh and colleagues documented that sCD36 in plasma was associated with circulating microparticles [32]. Therefore, we assumed that sCD36 might circulate in microparticles derived from Kupffer cells in those patients. Surprisingly, no expression of the CD36 protein on hepatocytes was found in the liver specimens of patients with CLD-C.

Recently, Miquilena-Colina and colleagues elucidated that the CD36 protein was largely expressed at the plasma membrane of hepatocytes in patients with HCV genotype 1 and hepatic steatosis, and patients with NASH [23], while the expression was predominantly located in the cytoplasm of hepatocytes in CH-C patients without hepatic steatosis. They described that weak expression of CD36 was also observed in the cytoplasm of scattered hepatocytes even in the normal liver. It is of interest that hepatic steatosis affected the localization of CD36 protein in the hepatocytes. However, these findings differed from our results that CD36 expression was not observed in the hepatocytes but the sinusoids. The reason for the discrepancy between our results and theirs remains unclear.

The present study also exhibited that the circulating sCD36 and oxLDL levels were obviously elevated in obese patients with CLD-C compared to those in NHCs. Nakhjavani and colleagues also confirmed significant higher level of oxLDL in patients with CH-C than in NHCs [33]. Increased serum oxLDL levels seemed to be derived from oxidative stress by persistent HCV infection. The serum sCD36 concentration was also elevated in proportion to the serum oxLDL level in patients with CLD-C. Moreover, the circulating sCD36 levels were associated with obesity in patients with CLD-C, which supports the previous evidence of a close correlation between the serum sCD36 level and BMI in patients with type 2 DM [24].

A previous article documented that the circulating sCD36 levels were associated with insulin resistance in non-diabetic obese patients [24]. It has been well established that the binding of oxLDL to CD36 results in the activation of c-Jun N-terminal kinase (JNK) and subsequent disruption of insulin signaling in both adipocytes and macrophages [34]. However, the current study did not reveal a significant correlation between the values of HOMA-IR and the circulating sCD36 levels in patients with CLD-C, although the serum sCD36 levels were higher in those patients than in NHCs.

A recent study proposed the circulating sCD36 level as a novel hallmark for liver damage in subjects with altered glucose tolerance [35]. However, we could not demonstrate a relation between the circulating sCD36 level and the liver damage in patients with CLD-C. The finding that most patients with liver cirrhosis develop severe insulin resistance, while the necroinflammation in those patients is attenuated may account for the lack of correlation between the serum sCD36 level and the severity of liver damage in patients with CLD-C.

It has been recently recognized that CD36 plays an indispensible role in the regulation of tissue uptake of free fat acid from plasma [36]. The expression of CD36 was decreased in adipose tissue, but increased in the liver. Such change in CD36 distribution might be involved in increased intrahepatic triglyceride content, and subsequently hepatic steatosis [36]. However, we could not provide the evidence that the circulating sCD36 level was associated with the severity of hepatic steatosis in the present study. Further examinations are required to clarify the discrepancy.

We postulated that the sCD36 levels are associated with infection by HCV genotype 1b, because the human SR-BI appeared to be a novel candidate receptor for the HCV of genotype 1b. However, the circulating sCD36 concentrations were independent of HCV genotypes in this study. The loads of HCV-RNA did not affect the serum sCD36 levels in those patients, either. These results may indicate that sCD36 levels are regulated by host factors rather than the viral factors themselves.

SR-BI has been well known as a key host factor for HCV entry by way of HCV-CD81 interaction [37]. The serum sCD36 levels were decreased by the PEG-IFN-based anti-viral treatments among patients with CH-C who achieved SVR. This result supported the finding by Murao and colleagues that exogenous IFN-α suppressed the expression of human SR-BI [38]. They demonstrated that the expression of human SRB-I was decreased through the inhibition of the signal transducer and activator of transcription (STAT) 1/STAT2 pathway. Therefore, the reduction of the circulating sCD36 level by antiviral treatments may imply the successful eradication of HCV. Likewise, this study also showed that the serum oxLDL levels tended to be decreased by antiviral treatments in patients with CH-C.

CD36 deficiency type 1 and type 2 were previously observed in 1.0% and 5.8% of healthy volunteers, respectively [39]. The present study revealed that the prevalence of CD36 deficiency type 2 in patients with CLD-C was approximately equivalent to that in NHCs, although flow cytometric analysis was performed in only 20 patients with CLD-C. The relationship between CD36 deficiency and insulin resistance remains controversial. One study revealed the association of CD36 deficiency with insulin resistance in patients with coronary heart diseases [21], while another study did not [40]. There was only one patient who fulfilled the category of CD36 deficiency type 2. Therefore, we could not elucidate the relationship between CD36 deficiency and insulin resistance in patients with CLD-C.

There are some limitations in this study. First, the number of flow cytometry tested for CD36 deficiency, and the number of paired samples to determine sCD 36 and oxLDL levels before and at the end of antiviral treatments, may be insufficient for the statistical analyses. However, a definite tendency was shown in each parameter.

Second, we could not compare hepatic expression of CD36 between patients with CLD and NHCs, because liver tissue specimens were not obtained from NHCs. Therefore, we speculated that the severity of hepatic CD36 expression was more extensive in obese patients with CLD-C than in NHCs through the result that the serum sCD36 levels were significantly higher in obese patients with CLD than in NHCs.

## Conclusion

We concluded that the serum sCD36 levels generally substituted for the severity of CD36 expression on the Kupffer cells in patients with CLD-C, and that serum sCD36 levels were dependent on obesity, but were neither associated with insulin resistance nor hepatic steatosis in those patients.

## Abbreviations

ALT: Alanine aminotransferase; BMI: Body mass index; CH-C: Chronic hepatitis C; CLD-C: HCV-related chronic liver disease; DM: Diabetes mellitus; HCV: Hepatitis C virus; HOMA-IR: Homeostasis model for assessment of insulin resistance; IRS-1: Insulin receptor substrate-1; NASH: Nonalcoholic steatohepatitis; NHC: Normal healthy control; oxLDL: Oxidized low-density lipoprotein; PEG-IFN: Pegylated interferon; sCD36: Soluble form of CD36; SR-BI: Scavenger receptor class B type I.

## Competing interests

The authors declare that they have no competing interest.

## Authors’ contributions

TH, JT, HM, AM, HY, KK and TM supplied sera and tissue specimens. RH and MU participated in the interpretation of pathological data. MI, HM, FG, MU and GY contributed to the technical supports. SS and TM provided the suggestion for this study. All authors read and approved the final manuscript.

## References

[B1] Rosen HR Clinical practice. Chronic hepatitis C infection N Engl J Med 2011 364 2429 2438 10.1056/NEJMcp1006613 21696309

[B2] Koike K Hepatitis C, as a metabolic disease: implication for the pathogenesis of NASH Hepatol Res 2005 33 145 150 10.1016/j.hepres.2005.09.023 16202646

[B3] Negro F Sanyal AJ Hepatitis C virus, steatosis and lipid abnormalities: clinical and pathologic data Liver Int 2009 S2 26 37 19187070 10.1111/j.1478-3231.2008.01950.x

[B4] Kohgo Y Ikuta K Ohtake T Torimoto Y Kato J Iron overload and cofactors with special reference to alcohol, hepatitis C virus infection and steatosis/insulin resistance World J Gastroenterol 2007 13 4699 4706 17729391 10.3748/wjg.v13.i35.4699 PMC4611191

[B5] Shintani Y Fujie H Miyoshi H Tsutusumi T Tsukamoto K Hepatitis C virus infection and diabetes: direct involvement of the in the development of insulin resistance Gastroenterol 2004 126 840 848 10.1053/j.gastro.2003.11.056 14988838

[B6] Poynard T Ratziu V McHutchison J Manns M Goodman Z Effect of treatment with peginterferon or interferon alpha-2b and ribavirin on steatosis in patients infected with hepatitis C Hepatol 2003 38 75 85 10.1053/jhep.2003.50267 12829989

[B7] Romero-Gomez M Del Mar VM Andrade RJ Salmeron J Diago M Insulin resistance impairs sustained response rate to peginterferon plus ribavirin in chronic hepatitis C patients Gastroenterol 2005 128 636 641 10.1053/j.gastro.2004.12.049 15765399

[B8] Lange CM Kutalik Z Morikawa K Bibert S Cerny A Serum ferritin levels are associated with a distinct phenotype of chronic hepatitis C poorly responding to pegylated interferon-alpha and ribavirin therapy Hepatol 2012 55 1038 1047 10.1002/hep.24787 22095909

[B9] Fartoux L Poujol-Robert A Guechot J Wendum D Poupon R Serfaty L Insulin resistance is a cause of steatosis and fibrosis progression in chronic hepatitis C Gut 2005 54 1003 1008 10.1136/gut.2004.050302 15951550 PMC1774632

[B10] Hung CH Wang JH Hu TH Chen CH Chang KC Insulin resistance is associated with hepatocellular carcinoma in chronic hepatitis C infection World J Gastroenterol 2010 16 2265 2271 10.3748/wjg.v16.i18.2265 20458764 PMC2868220

[B11] Koike K Moriya K Metabolic aspects of hepatitis C viral infection: steatohepatitis resembling but distinct from NASH J Gastroenterol 2005 40 329 336 10.1007/s00535-005-1586-z 15868369

[B12] Himoto T Yoneyama H Deguchi A Kurokohchi K Inukai M Insulin resistance derived from zinc deficiency in non-diabetic patients with chronic hepatitis C Exp Ther Med 2010 1 707 711 22993593 10.3892/etm_00000109 PMC3445882

[B13] Himoto T Yoneyama H Kurokohchi K Inukai M Masugata H Selenium deficiency is associated with insulin resistance in patients with hepatitis C virus-related chronic liver disease Nutr Res 2011 31 829 835 10.1016/j.nutres.2011.09.021 22118753

[B14] Febbraio M Hajjar DP Silverstein RL CD36: a class B scavenger receptor involved in angiogenesis, atherosclerosis, inflammation, and lipid metabolism J Clin Invest 2001 108 785 791 11560944 10.1172/JCI14006 PMC200943

[B15] Cai L Wang Z Ji A Meyer JM van der Westhuyzen DR Scavenger receptor CD36 expression contributes to adipose tissue inflammation and cell death in diet-induced obesity PLoS One 2012 7 e36785 10.1371/journal.pone.0036785 22615812 PMC3353961

[B16] Tuomisto TT Riekkinen MS Viita H Levonen AL Yla-Herttuala S Analysis of gene and protein expression during monocyte-macrophage differentiation and cholesterol loading-cDNA and protein array study Atherosclerosis 2005 180 283 291 10.1016/j.atherosclerosis.2004.12.023 15910854

[B17] Griffin E Re A Hamel N Fu C Bush H A link between diabetes and atherosclerosis: glucose regulates expression of CD36 at the level of translation Nat Med 2001 7 840 846 10.1038/89969 11433350

[B18] Sampsom MJ Davies IR Braschi S Ivory K Hughes DA Increased expression of a scavenger receptor (CD36) in monocytes from subjects with type 2 diabetes Atherosclerosis 2003 167 129 134 10.1016/S0021-9150(02)00421-5 12618277

[B19] Zhou J Febbraio M Wada T Zhai Y Kuruba R Hepatic fatty acid transporter cd36 is a common target of LXR, PXR, and PPARγ in promoting steatosis Gastroenterol 2008 134 556 567 10.1053/j.gastro.2007.11.037 18242221

[B20] Koonen DPY Jacobs RL Febbraio M Young ME Soltys CLM Increased hepatic CD36 expression contributes to dyslipidemia associated with diet-induced obesity Diabetes 2007 56 2863 2871 10.2337/db07-0907 17728375

[B21] Miyaoka K Kuwasako T Hirano K Kozai S Yamashita S Matsuzawa Y CD36 deficiency associated with insulin resistance Lancet 2001 357 686 687 10.1016/S0140-6736(00)04138-6 11247555

[B22] Scarselli E Ansuini H Cerino R Roccasecca RM Acali S The human scavenger receptor class B type 1 is a novel candidate receptor for the hepatitis C virus EMBO J 2002 21 5017 5025 10.1093/emboj/cdf529 12356718 PMC129051

[B23] Miquilena-Colina ME Lima-Cabello E Sanchez-Campos S Garcia-Mediavilla MV Fernandez-Bermejo M Hepatic fatty acid translocase CD36 upregulation is associated with insulin resistance, hyperinsulinemia and increased steatosis in non-alcoholic steatohepatitis and chronic hepatitis C Gut 2011 60 1394 1402 10.1136/gut.2010.222844 21270117

[B24] Handberg A Levin K Hojlund K Beck-Nielsen H Identification of the oxidized low-density lipoprotein scavenger receptor CD36 in plasma: a novel marker of insulin resistance Circulation 2006 114 1169 1176 10.1161/CIRCULATIONAHA.106.626135 16952981

[B25] Handberg A Norberg M Stenlund H Hallmans G Attermann J Eriksson JW Soluble CD36 (sCD36) clusters with markers of insulin resistance, and high sCD36 is associated with increased type 2 diabetes risk Clin Endocrinol Metab 2010 95 1939 1946 10.1210/jc.2009-2002 20139232

[B26] Lau JY Davis GL Kinffen J Qian KP Urdea MS Significance of serum hepatitis C virus RNA levels in chronic hepatitis C Lancet 1993 341 1501 1504 10.1016/0140-6736(93)90635-T 8099380

[B27] Sandres-Saune K Abravanel F Nicot F Peron JM Alric L Detection and quantitation of HCV RNA using real-time PCR after automated sample processing J Med Virol 2007 79 1821 1826 10.1002/jmv.21003 17935166

[B28] Simmonds P Alberti A Alter HJ Bonino F Bradley DW A proposed system for nomenclature of hepatitis C viral genotypes Hepatology 1994 19 1321 1324 10.1002/hep.1840190538 8175159

[B29] Ichida F Tsuji T Omata M Ichida T Inoue K New Inuyama classification: new criteria for histological assessment of chronic hepatitis Int Hepatol Commun 1996 6 112 119 10.1016/S0928-4346(96)00325-8

[B30] Brunt EM Janney CG Bisceglie D Neuschwander-Tetri BA Bacon BR Nonalcoholic steatohepatitis: a proposal for grading and staging the histological lesions Am J Gastroenterol 1999 94 2467 2474 10.1111/j.1572-0241.1999.01377.x 10484010

[B31] Imajo K Fujita K Yoneda M Nozaki Y Ogawa Y Hyperresponsivity to low-dose endotoxin during progression to nonalcoholic steatohepatitis in regulated by leptin-mediated signaling Cell Metab 2012 16 44 54 10.1016/j.cmet.2012.05.012 22768838

[B32] Alkhatatbeh MJ Mhaidat NM Enjeti AK Lincz LF Thorne RF The putative diabetic plasma marker, soluble CD36, is non-cleaved, non-soluble and entirely associated with microparticles J Thromb Haemost 2011 9 844 851 10.1111/j.1538-7836.2011.04220.x 21276198

[B33] Nakhjavani M Mashayekh A Khalilzadeh O Asgarani F Morteza A Oxidized low-density lipoprotein is associated with viral load and disease activity in patients with chronic hepatitis C Clin Res Hepatol Gastroenterol 2011 35 111 116 10.1016/j.clinre.2010.11.001 21809486

[B34] Kennedy DJ Kuchibhotla S Westfall KM Silverstein RL Morton RE Febbraio M A CD36-dependent pathway enhances macrophage and adipose tissue inflammation and impairs insulin signaling Cardiovasc Res 2011 89 604 613 10.1093/cvr/cvq360 21088116 PMC3028977

[B35] Fernandez-Real JM Handberg A Ortega F Hojlund K Vendrell J Richart W Circulating soluble CD36 is a novel marker of liver injury in subjects with altered glucose torelance J Nutr Biochem 2009 20 477 484 10.1016/j.jnutbio.2008.05.009 18789673

[B36] Fabbrini E Sullivan S Klein S Obesity and Nonalcoholic fatty liver disease: biochemical, metabolic, and clinical implications Hepatol 2010 51 679 689 10.1002/hep.23280 PMC3575093 20041406

[B37] Zeisel MB Koutsoudakis G Schnober EK Haberstroh A Blum HE Scavenger receptor class B type I is a key host factor for hepatitis C virus infection required for an entry step closely linke to CD81 Hepatol 2007 46 1722 1731 10.1002/hep.21994 18000990

[B38] Murao K Imachi H Yu X Cao WM Nishiuchi T Interferon a decreases expression of human scavenger receptor class B1, a possible HCV receptor in hepatocytes Gut 2008 57 664 671 10.1136/gut.2006.111443 17998316

[B39] Yanai H Chiba H Fujiwara H Morimoto M Abe K Phenotype-genotype correlation in CD36 deficiency type I and II Thromb Haemost 2000 84 436 441 11019968

[B40] Furuhashi M Ura N Nakata T Shimamoto K Insulin sensitivity and lipid metabolism in human CD36 deficiency Diabetes Care 2003 26 471 474 10.2337/diacare.26.2.471 12547883

